# The typical and atypical development of empathy: How big is the gap from lab to field?

**DOI:** 10.1002/jcv2.12136

**Published:** 2023-01-24

**Authors:** Chiara Bulgarelli, Emily J. H. Jones

**Affiliations:** ^1^ Centre for Brain and Cognitive Development Birkbeck University of London London UK; ^2^ Department of Medical Physics and Biomedical Engineering University College London London UK

**Keywords:** antisocial behaviours, callousness‐unemotional traits, emotions, empathy, naturalistic observations, new methodologies, toddlerhood

## Abstract

**Background:**

Empathy‐understanding and sharing someone else's feelings‐is crucial for social bonds. Studies on empathy development are limited and mainly performed with behavioural assessments. This is in contrast to the extensive literature on cognitive and affective empathy in adults. However, understanding the mechanisms behind empathy development is critical to developing early interventions to support children with limited empathy. This is particularly key in toddlerhood, as children transition from highly scaffolded interactions with their parents and towards interactions with their peers. However, we know little about toddlers' empathy, in part due to the methodological constraints of testing this population in traditional lab settings.

**Methods:**

Here, we combine naturalistic observations with a targeted review of the literature to provide an assessment of our current understanding of the development of empathy in toddlerhood as it is expressed in real‐world settings. We went into toddlers' typical habitat, a nursery, and we performed 21 h of naturalistic observations of 2‐to‐4‐year‐olds. We then reviewed the literature to evaluate our current understanding of the mechanisms that underpin observed behaviours.

**Results:**

We observed that (i) emotional contagion, possibly a primitive form of empathy, was observed at the nursery, but rarely; (ii) older toddlers often stared when someone cried, but there was no clear evidence of shared feelings; (iii) teacher and parent scaffolding might be paramount for empathy development; (iv) as some atypical empathic reactions can be observed from toddlerhood, early interventions could be developed. Several competing theoretical frameworks could account for current findings.

**Conclusions:**

Targeted studies of toddlers and their interaction partners in both controlled and naturalistic contexts are required to distinguish different mechanistic explanations for empathic behaviour in toddlerhood. We recommend the use of new cutting‐edge methodologies to embed neurocognitively‐informed frameworks into toddlers' natural social world.

**Key points**

- Studying toddlerhood empathy facilitates prodromal intervention for emerging callous‐emotional traits, but mechanistic empirical studies are limited by the challenge of lab testing.
- We used real‐world observations of toddlers in nursery to contextualise and evaluate the ecological validity of the empirical literature.
- Behavioural signs of emotional contagion were observed very rarely. Is this behaviour really a primitive and foundational form of empathy?
- Staring at a classmate in distress was common, but with no clear behavioural signs of sharing sad feelings (e.g. a sad expression). Embedding neuroimaging may reveal whether this reflects cognitive or affective empathy, or whether this provide opportunities for caregiver scaffolding that can be leveraged in interventions.
- Early‐emerging a typicality in empathy can be reflected in aggression towards peers; competing theoretical models that could underpin mechanistic interventions could be tested through new neuroimaging techniques in virtual reality environments.

## INTRODUCTION

Empathy is understanding and sharing someone else's feeling, and it is the crucial foundation of social bonds (Singer & Lamm, 2009). Humans are social creatures and being empathic has been positively associated to cooperation (Rumble et al., 2010), group formation (Anderson & Keltner, 2002) and intergroup relations (Vanman, 2016). Understanding the early development of empathy is clinically relevant because lack of early empathy characterises children with callous‐unemotional (CU) traits (Shirtcliff et al., 2009; Viding et al., 2012), who are at high risk of developing severe and persistent antisocial behaviour and conduct problems^1^ (Frick et al., 2014). This represents the most common reason for referral to mental health services in childhood (NICE and SCIE, 2017; Viding et al., 2012) and a substantial cost for society, as supporting a child with antisocial behaviours into adulthood can cost up to 10 times more than a typically developing child (Scott et al., 2001). As early onset of antisocial behaviour is predictive of worst outcome in adulthood (McGee et al., 2011), early identification provides opportunities for early intervention to support later trajectories.

In adults, there is an extensive literature documenting the neural and cognitive mechanisms underlying empathic behaviours (for example see Bernhardt & Singer, 2012; Decety & Jackson, 2006; Shamay‐Tsoory, 2011). General frameworks identify two main components of empathy, an affective one (i.e. experiencing a feeling that is alike the one perceived to be felt by the other) and a cognitive one (i.e. understanding the other's emotional state) (Bird & Viding, 2014; Gonzalez‐Liencres et al., 2013). Adult fMRI studies, where participants are shown pictures or videos of others in distress (to elicit affective empathy) or asked to think about others' emotions (to elicit cognitive empathy), suggested that cognitive and affective empathy are supported by different neural networks; while the dorsolateral frontal cortex and the bilateral temporo‐parietal junction have been associated to cognitive empathy, affective empathy is supported by ventromedial frontal cortex and superior temporal gyrus (Shamay‐Tsoory, 2011). However, how and when these two components mature is poorly understood.

In research on the development of empathy, terminology and conceptual frameworks are often used imprecisely. Many studies conflate empathy with the development of broader related social skills (such as prosocial behaviours, emotion recognition) (see Box 1 for a glossary).^2^ However, to progress it is important to focus empirical works on the study of empathy per se, as helping other children might not necessarily entail sharing their feelings. Further, debate remains over whether phenomena like contagious crying reflect affective empathy, or whether they reflect personal distress (aversive reactions to an irritating stimulus that serves to allow the newborn to compete for attention and thus have the opposite behavioural drive to affective empathy) (Decety & Holvoet, 2021).

**BOX 1 Glossary of empathy**

Empathy research has suffered from confusion around its terms. Defining a common glossary of such terminology is critical to progression in the field.

- Emotional contagion: automatically generated behaviours and feelings that do not imply understanding other's feelings, and do not require a clear self‐other distinction; could reflect early affective empathy or personal distress
- Personal distress: negative reaction to cue associated with others' emotional state (e.g. finding the acoustic properties of crying irritating, being overwhelmed with self‐oriented stressful or sad feelings when another person is sad)
- Affective or Emotional Empathy: sharing someone else's feeling and experiencing the same arousal content, but experienced as an “other‐oriented” feeling. This is possible only once infants have developed self‐other differentiation.
- Sympathy: experiencing concern and sorrow in response to negative impacts on other's wellbeing
- Cognitive Empathy: understanding someone else's feelings and the underlying reasons
- Empathy: sharing and understanding someone else's feeling
- Theory of Mind: understanding someone else's intentions and thoughts

Toddlerhood is a critical age to study the development of empathy, as children emerge from heavily scaffolded interactions with their caregiver and into interactions with their peers, where interactions are heavily dependent on their own skills. At around 2 years toddlers are able to distinguish whether emotions originate from themselves or others (Amsterdam, 1972; Bulgarelli et al., 2019). However, many laboratory studies focus only on school‐age children, when antisocial behaviour can be more clearly identified (Viding & McCrory, 2019). One key reason is that toddlerhood is a difficult age to test in laboratories, as children at this age struggle to comply with lab testing rules, such as sitting still and following instructions. Moreover, in traditional lab studies toddlers' behaviours in social tasks might be artificial and distinct from what might be observed in a more relaxed environment. Therefore, to develop efficient experimental approaches to explore the development of empathy, to suggest new research questions, and to discuss whether our limited knowledge on empathy in toddlerhood is accurate, we began by observing toddlers in the “real world”, a nursery class, where toddlers feel confident and not under the experimenter's magnifying glass.

In this work, we discuss insights from 21 h of empirical observations of empathy in toddlers as a context to evaluating the most recent developmental literature on empathy in the first 3 years of life (for empathy in childhood see for example Decety & Holvoet, 2021; Frick & Kemp, 2021). Through comparison of existing literature with our observations, we probe the ecological validity of our current models of empathy development, and future directions for ecologically‐informed neuroimaging studies.

## HOW FAR IS LAB RESEARCH FROM THE “REAL WORLD”? INSIGHTS BASED ON NATURALISTIC OBSERVATIONS OF 2‐TO‐4‐YEAR‐OLDS IN A NURSERY

We carried out 21 h of observations in the toddler and preschool classes of a nursery in North London (see supporting information). Table 1 summarises the typical patterns of behaviour observed, of which the most relevant to empathy were contagious crying in younger children and increased attentiveness to children displaying distress in the older children, who also showed more cooperative play, pretend play and emotion language (Box 2). Atypical empathic behaviour was also observed, indicating the relevance of this age range to understanding emerging callous‐unemotional traits (Box 3).

**BOX 2 Reactions to a classmate crying**

**FIGURE 1:**
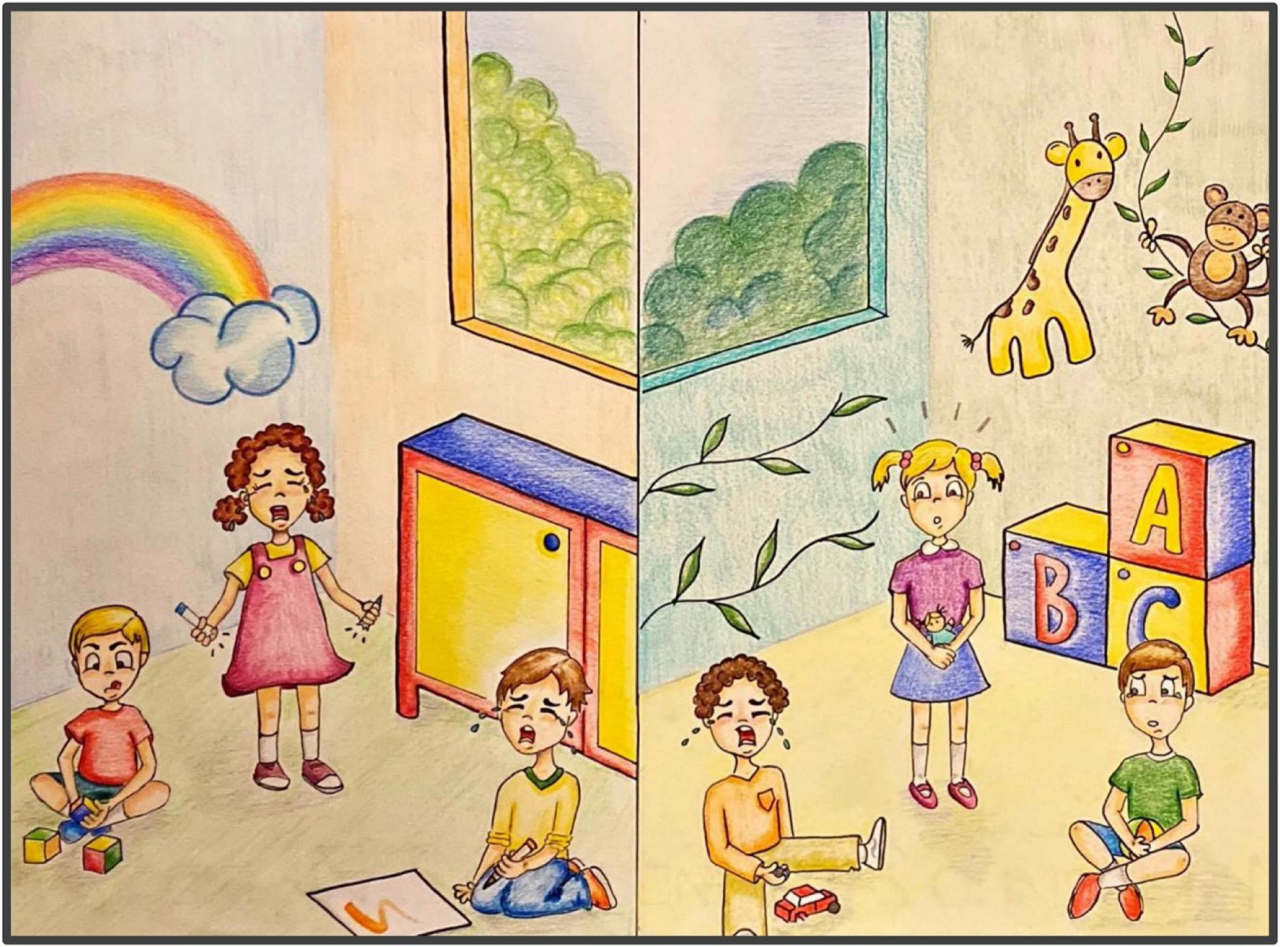
Graphical representation of the different reactions to a toddler crying in 2‐to‐3‐year‐olds, on the left, and 3‐to‐4‐year‐olds, on the right. On the left panel, younger children showed indifference, such as the child on the left playing with blocks (red t‐shirt) or emotional contagion, such as the child crying on the right (yellow t‐shirt), while their classmate was crying in the centre of the scene (pink dress). On the right panel, older children showed signs of personal distress, such as the child standing in the middle of the scene (pink t‐shirt) and one on the right (green t‐shirt) staring puzzled in front of their classmate crying (child seated with orange t‐shirt).

Observing a toddler crying was quite common in both classes, triggered either by personal discomfort (e.g. missing their mum, being tired) or by someone else's behaviour (e.g. being hurt, having a toy extorted). Reactions to someone crying was different in the two age groups (Figure 1). The experimenter noted several episodes in which 2‐to‐3‐year‐olds did not react to this, continuing their activity, even if the child crying was physically close to them. However, there was an episode where a couple of young toddlers started crying after looking at a classmate crying. This is an extract for the experimenter's notes in the 2‐to‐3‐year‐olds class:

M(3) cries very loudly in a corner, he throws himself on the floor, because he wet himself, but he doesn't want to be changed. A teacher next to him is trying to console him, but M(3) keeps crying. At first, the other children in the class do not react to M(3) crying, even if they are close to him. After a couple of minutes, F(2) goes next to M(3) crying, she sits next to him and starts crying. After a minute, F(2) stopped crying autonomously, leaves and goes playing in the sandpit, without showing any signs of distress anymore. M(3) is still crying very loudly, F(3) comes in front of him, and she stares at him. After a few seconds, F(3) starts crying, while keeping staring at M(3). Then F(3) leaves and stops crying autonomously. M(3) is still crying very uproariously, M(3) comes in front of him, he stares at him for a few seconds and then he leaves. The teacher is finally able to calm M(3) down and change his clothes. While all this is happening, there are 5/6 other 2‐to‐3‐year‐olds in the class that did not react to M(3) crying, and continued the activity they were focusing on.

In the 3‐to‐4‐year‐olds class, there were many instances where the experimenter observed children staring at someone else crying. Only in one episode a toddler hugged a classmate crying, following his teacher's example. None of the 3‐to‐4‐year‐olds was observed crying in response to a classmate's crying. Here there are some extracts for the experimenter's notes in the 3‐to‐4‐year‐olds class:

- *F(4) is crying, the teacher gets close to console her. 3 children (2F(4) 1M(3)) go close to F(4), they stare at F(4) crying, they all show a sad face.*
- *On the sand‐pit, M(4) throws some sands under M(4)'s t‐shirt, who starts crying, saying “I don't like it”. M(4), who threw the sand, F(4), and M(3) stare at M(4) crying but do nothing. F(4) has a sad face.*
- *F(4) hits strongly M(4), the teacher gets close to console him. Another F(4) follows the teacher and stares at M(4) crying. Next to M(4) crying, there are other F and M, but they do not react.*
- *M(4) cries very loudly as he hurt his finger, F(3) stares at him.*
- *F(3) cries as she hurt herself with a wooden box full of toys, the teacher next to her hugs her. F(4) stares at F(3) crying.*
- *F(4) cries and the teacher hugs her. M(3), who was with the teacher, smiles and hugs F(4) and the teacher.*
- *During the group activity, F(3) starts crying very loudly and she leaves the group, trying to get the others' attention. A teacher goes to her to console her, F(3) follows the teacher, and she stares at F(3) crying.*
- *F(4) hits F(3) on her neck, F(3) starts crying. Another F(4) comes closer and stares at F(3) crying.*

**BOX 3 Reduced empathic reactions in a 4‐year‐old child**

**FIGURE 2:**
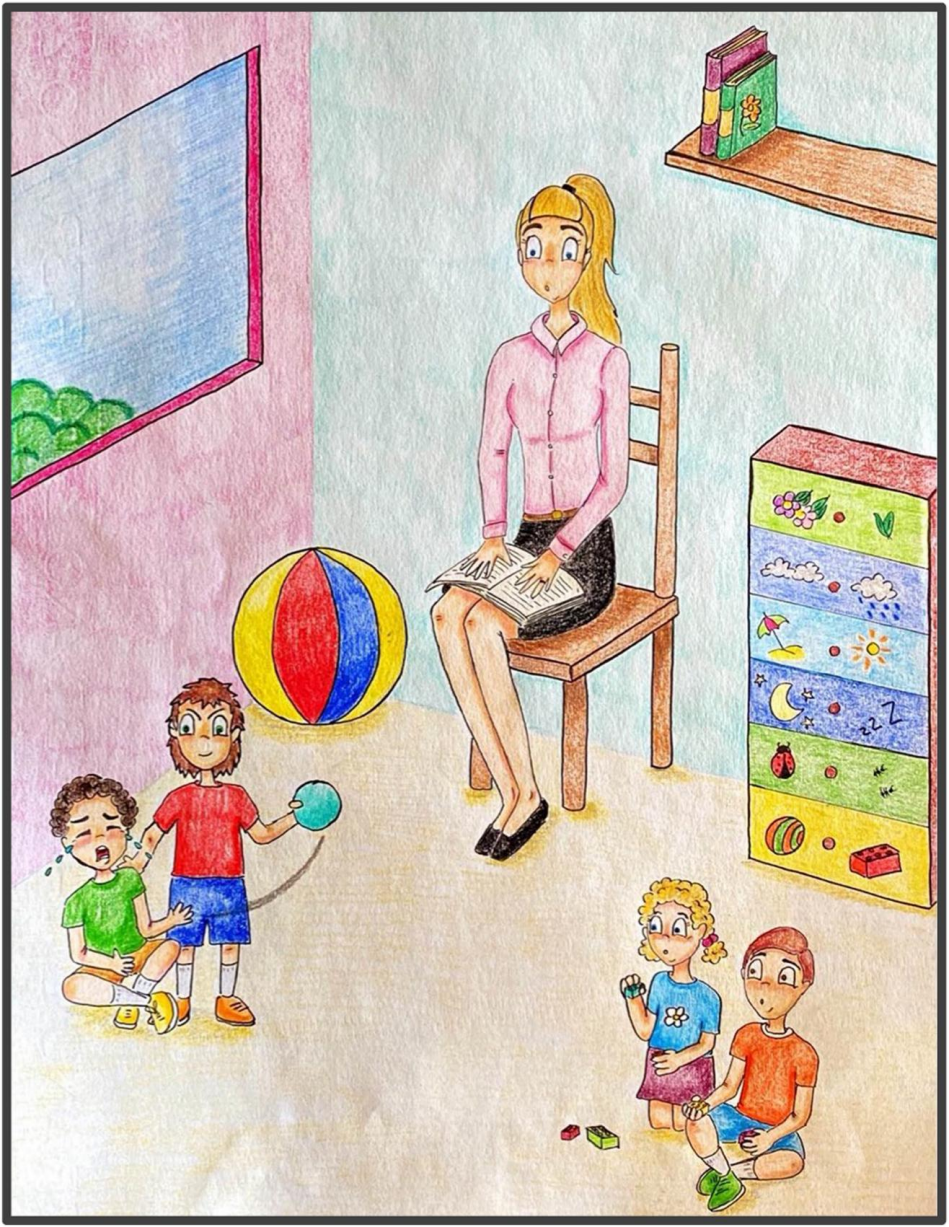
Graphical representation of a child with possibly poor empathic skills, evidenced by a non‐reaction to others' distress.

One 4‐year‐old child showed clear disruptive behaviours towards other classmates, without any sign of emotional contagion or personal distress. Instead, the child often hurt other children without showing guilt or remorse (Figure 2). This is an extract from the experimenter's notes, where the child under observation is marked with X(4)*:

- X(4)* hits M(4) quite heavily on his head, and he starts crying. While other children go towards M(4), X(4)* smiles and runs away (this happened at least 3 times during the observations). X(4)* moves away while seeing that a teacher looking at what happened goes to M(3) to console him. X(4)* calls the teacher to get her attention, and laugh.
- F(3) and F(4) are playing with trains. X(4)* goes towards them and starts playing with trains. X(4)* steals a train from F(3)'s hands, and says “you cannot play with us”. F(3) starts crying and goes away, F(4) goes away and X(4)* keeps playing by herself.

**TABLE 1:** Summary of the most frequently observed behaviours

Behaviour	2‐to‐3‐years	3‐to‐4‐years
Playing with someone else	Most of the time playing individually	Individual playing but also playing in dyads or groups
Nature of the interactions	Physical interactions most of the time (e.g. hurting another toddler, or stealing a toy from someone else's hand)	Physical interactions but also cooperation and playing together (e.g. building a tower, building a sandcastle, colouring on the same paper)
Talk/express themselves	Cannot express themselves and speak very well	Clear speech, some of them use abstract words (facilitated by the teachers, (“I can see you are angry”)
Pretend play	Almost absent	Often present
Reaction to someone else's crying	Either no reaction (∼60%) or crying themselves too (∼40%) (consistent with emotional contagion)	Staring at the child crying, rarely with a sad face (∼15%) (Box 2)

**TABLE 2:** Methods used to assess affective and cognitive empathy in the studies discussed in this review

	Modalities			
	Behavioural	Questionnaires	Neuroimaging	Physiological
Affective empathy	Expressions of sobering, brow furrows, sadness sympathetic, fearfulness, anger–aggression in response to seeing their mother in pain (Volbrecht et al., 2007). Facial, vocal, or gestural–postural expressions in response to seeing the experimenter in pain (Knafo, 2009). Attribution of emotional states, facial affective expressions, matching facial expressions during an emotionally salient story (Knafo, 2009). Signs of compassions in response to a distressed playmate (Bischof‐Köhler, 2012). Facial and vocal gestures, bodily agitation freezing, crying in response to a doll crying (Nichols, 2015). Signs of anxiety or discomfort in response to a crying doll (McHarg et al., 2019). Coding of comfort seeking, personal distress and empathic concern towards the experimenter's pain (Noten, 2020).	*Questionnaire of Cognitive and Affective Empathy (QCAE)* (Reniers, 2011) measuring the trait parent empathy (Decety, 2018) *Emotional Symptoms sub‐ scale of the Strengths and Difficulties Questionnaire* (Goodman, 1997) maternal report on child abilities (Knafo, 2009).	Auditory and visual scenarios of with emotionally relevant stories with a positive or negative end (Brink, 2011). N2 ERP component measured while toddlers looked at others in pain and were asked how much pain the other feels and how sorry they feel for the other (Decety, 2018).	Cardiac pulsation measured while toddlers looked at the experimenter in pain (Noten, 2020).
Cognitive empathy	Emotion recognition of facial expression and vocal affects (Stevens et al., 2001; BLAIR, 2001). Approaching and helping behaviours in reponse to seeing their mother in pain (Volbrecht et al., 2007). Helping behaviours in reponse to distressed playmate (Bischof‐Köhler, 2012). Questions about character's feeling in emotional stories (Bensalah, 2016). Labelling of emotional state and helping behaviours in response to a crying doll (McHarg et al., 2019).		Auditory and visual scenarios with a logical or an illogical end (Brink, 2011)	

## HOW WELL DOES THE SCIENTIFIC LITERATURE EXPLAIN REAL‐WORLD PATTERNS OF EMPATHY IN TODDLERS?

### Emotional contagion and foundations of empathy in infants

During our observations of toddlers in their own “habitat”, we observed that those younger than 3 years primarily showed indifference and sometimes what appeared to be emotional contagion to a classmate crying. Developmental psychologists generally consider that young infants are capable of affect sharing and emotional contagion. Unlike affective empathy, in which one shares other's feelings while understanding that they are separated from one's own, emotional contagion is considered to represent automatically generated behaviours and feelings that do not imply understanding other's feelings, and do not require a clear self‐other distinction^3^ (Decety et al., 2012; Gonzalez‐Liencres et al., 2013). The most common example of this is contagious crying. Measuring behavioural of physiological reactions to other infants' crying, as indicated for example, by increased sucking rate or increased heart rate, is the most common method to assess emotional contagion in infancy (Geangu et al., 2010). These reactions indicating emotional contagion have been widely documented in newborns as well (Dondi et al., 1999; Martin & Clark, 1982; Sagi & Hoffman, 1976; Simner, 1971), but lower in depressed mothers' newborns (Field et al., 2007).

Many consider emotional contagion as a primitive form of empathy and fundamental to prepare the infant to react to someone else's emotional state (Geangu et al., 2011; McDonald & Messinger, 2011). Given its adaptive advantage for social species, emotional contagion has been observed also in non‐human animals. For example, mice produced writhing behaviours when looking at another mouse in pain, especially if they experienced the same pain too (Langford et al., 2006), and they showed a greater fear response if the mouse in pain was “socially” related to themselves compared to a stranger‐mouse (Jeon et al., 2010). Emotional contagion might then be an old phylogenetic behaviour that appears ontogenically in infants but is later superseded by more cognitively enriched forms of empathy. Evolutionarily speaking, empathy might have developed to support caring behaviours towards our kin, to foster the advancement of those more similar to us (Gonzalez‐Liencres et al., 2013).

An alternative to the nativist perspective is that the spontaneous tendency to copy others (Chartrand & Bargh, 1999), might induce emotional contagion (Heyes, 2018; Prochazkova & Kret, 2017). In this framework, *showing* some emotions promote their *feeling*. Mimicking someone's facial expression can help understand and categorising the emotional expression mimicked, by sharing activations in the corresponding brain regions (Buck, 1980). As there is evidence that mimicry arises very early in infancy and is triggered by social ostensive cues (de Klerk et al., 2018), mimicking others might support associative learning of the links between an infant's emotion and their parent's. More general forms of associative learning (between the sound of own cries and negative affect) have also been posited as an explanation for both contagious crying and affective empathy more broadly (Heyes, 2018). It is worth mentioning that the nativist and the learning accounts should not necessarily be viewed in contrast. In fact, it is possible that while a predisposition towards others' emotions and pain seems to be present from early on, the environment might promote and shape these reactions soon after. This nature/nurture interaction is not atypical in infant development, especially in the social domain (for example see Santamaria et al., 2020).

The idea that emotional contagion is a precursor or early form of affective empathy is not universally accepted. Others proposed that infants' contagious crying might have the scope to obtain the caregiver's attention, therefore functional to one's own survival (Campos & Barrett, 1984), or merely represent a distress response to acoustic features of the cries (Ruffman et al., 2017). Consistent with this, it has been shown that infants younger than 1 year rarely showed distress to someone else crying, as indicated by facial grimace, whimpering, or crying (Roth‐Hanania et al., 2011), and that mothers' stressful state can induce distress in their own infants (Waters et al., 2014). Furthermore, contagious crying becomes much less common by 5 months when infants stop crying in response to another infant crying (Martin & Clark, 1982). Does this behaviour then reappear later in toddlerhood and if so, is this underpinned by similar or different mechanisms? Does contagious crying in toddlers represent sharing an other‐oriented emotional state (feeling sad because someone else is sad), or finding the attention or noise of another toddler crying unpleasant or overwhelming (personal distress) (Martin & Clark, 1982; Sagi & Hoffman, 1976; Simner, 1971)? Conceptually and functionally, these experiences are different because they invoke positive/affiliated versus negative/exclusionary attitudes towards the interaction partner, and only the former would be considered affective empathy.

What are the mechanisms that link these experiences of shared distress to the emergence of empathy? In the field, we observed that episodes of emotional contagion in younger toddlers happened rarely and were not present in older toddlers, while (apparent) indifference was more predominant. Fewer instances of some behaviours in the real‐world than those from empirical works have been documented elsewhere, such as for face looking or gaze following (Franchak et al., 2011). This poses a challenge for theoretical models that lay emotional contagion as a foundation from which affective and cognitive empathy emerge. If emotional contagion is primarily present under the age of 5 months, with rare examples of continuation into toddlerhood, how does this form the foundation of affective and cognitive empathy skills that are not clear apparent until later childhood? In this respect, it is important to highlight that all the works that investigated emotional contagion or distress in toddlers assessed these behaviours towards adults or a doll (see Box 4). We found no studies that assessed emotional contagion or empathic reactions during toddler‐toddler interactions, which would more realistically resemble what we observed in nursery. This is also contrast with the literature on emotional contagion where newborns are tested in response to another newborn/doll crying, and is critical gap in the literature given associative learning accounts would predict that empathic reactions should be larger towards similarly‐aged children (Heyes, 2018). Further, most of the literature focuses on negative events and affect sharing, but positive affect sharing (i.e. shared smiles), which are frequent in a young infant's social life, should also be foundational for the development of empathy and should elicit stronger reactions under associative learning accounts.

**BOX 4 Methods to assess affective and cognitive empathy**

Table 2 reports the methods to assess affective and cognitive empathy of the studies cited in this work.

### The emergence of empathy in toddlerhood

**FIGURE 3:**
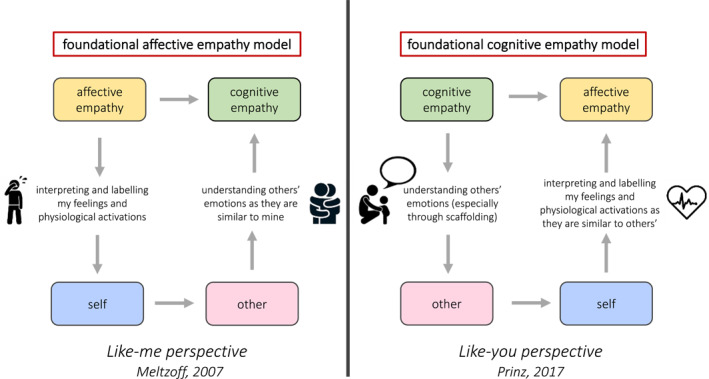
Graphical representation of the two possible models of the development of empathic components, in reference to the two models of the development of the sense of self.

A central question is the degree to which cognitive and affective empathy emerge in parallel, or sequentially, and toddlerhood is a critical window for this (Decety & Holvoet, 2021). In the nursery, we observed a transition from emotional contagion to watchful interest in negative emotion; does this reflect early cognitive or affective empathy (or neither)? Many would interpret the staring at someone crying as an indicator of distress (and so of affective empathy). However, looking at the other crying might help the toddler to understand the situation and therefore label other's emotion (cognitive empathy). Embedding neuroimaging or arousal recordings into naturalistic settings may be one way to determine the relative influence of cognitive or affective processes. This is critical to differentiating competing theories. Most frameworks posit that cognitive empathy matures on the root of affective empathy, which might either be innate (Decety & Holvoet, 2021) or learnt from others through associative learning (Heyes, 2018) (Figure 3). Signs of cognitive empathy have been found only in apes (indicated for example by helping behaviours) and humans, suggesting that these skills required more advanced brain structures and cognitive substrates (Edgar et al., 2012; Frick & Kemp, 2021). In a *foundational affective empathy model*, children begin by experiencing another's emotions through emotional contagion, then learn that these feelings are related to another person as they develop a self‐other distinction, and then use their emerging knowledge of the labels for their own internal experiences to label how another is feeling (cognitive empathy). However, as most of this evidence are limited on studies that assessed affective empathy based on reaction to another infant or a doll crying or in distress, to date there is no clear evidence of shared feelings where personal distress has been ruled out before the 2nd year of life. This is consistent with our observations in the nursery in which often there were no clear behavioural signs of shared other‐oriented feelings (e.g. sad expressions). However, we have to acknowledge that here we could only assess *seen* manifestation of emotions, without any indication of how the toddlers actually *felt*. Current data fits equally with a model in which cognitive empathy is foundational to true other‐oriented empathy, with the parallel but separate operation of emotional contagion through personal distress. By observing, mimicking, and labelling others' emotional reactions, and learning to understand and give a name to ones' own internal feeling and arousal, one may come to share another's feelings (*foundational cognitive empathy model*; Figure 3). In fact one could argue that there is actually considerable evidence that children understand at least some aspects of how someone else is feeling before they share the same feeling (for eample see O’Brien et al., 2011). It is plausible to hypothesise that toddlers learn (possibly supported by carers' scaffolding) to determine whether another child is sad, and they learn to help or cuddle him. Only after, toddlers might understand which of their own physiological activations and feelings are associated to sadness, likely supported by modelling, labelling, or mimicry. Pivotal next steps of research on empathy development should provide further evidence in support of either of these two frameworks.

Interestingly, these models relate to ongoing debates around the emergence of the self‐other distinction (Kampis et al., 2021); do we understand others because we have a clear sense of self (Meltzoff, 2007) or does the self arise from understanding others (Prinz, 2017)? These frameworks can only be distinguished through empirical data.

*Distinguishing self and other:* There is a key turning point in the development of some social skills in the second year of life, when infants mature the ability to distinguish between themselves and others (Amsterdam, 1972; Bulgarelli et al., 2019). For emotional contagion to become true affective empathy, infants need not only have developed self‐other differentiation, but also the ability to clearly differentiate between their own emotions and the feeling elicited by other's emotions. Consistent with this, it has been shown that 24‐month‐olds but not 12‐month‐olds showed behaviourally‐coded interest and concern to a doll crying (Nichols et al., 2015). Moreover, there was a strong correlation between empathic behaviours towards another child in distress and self‐other distinction in a sample of 2‐year‐old infants, even when correcting for age (Bischof‐Köhler, 2012). One limitation is that studies that assessed empathy in toddlers focused mainly on personal distress and reactions to negative events, that is, an infant crying (for example see McHarg et al., 2019) as indicators of affective empathy. Similarly, during our observations in nursery, the experimenter did not observe any children reacting to another child's positive emotions. There were some instances in which children were laughing together while playing, or episodes of reciprocal smiling, but we do not consider these as proper empathic reactions. As we observed empathy driven by other's *negative* emotions (reactions to someone else crying or in distress), similarly we think that empathy driven by other's *positive* emotions should be assessed by looking at toddlers' reaction to their classmate happy and joyful state, without necessarily being primarily involved in that emotional state. While this was hard to be picked up during our naturalistic observations, it might be that by using more sophisticated experimental designs, empathic reactions towards other's positive feelings, and not only towards negative ones, could be assessed. However, it might also be that these responses developed later than empathic reactions towards other's negative feelings. This might be seen as “negativity bias”—that is, the tendency to have a greater reaction for negative rather than positive emotions and events—which has been hypothesised to be present from early in life (Vaish et al., 2008). Therefore, one may think that the first empathic reactions arise for others' negative emotions (i.e. sadness, fear, anger) and then extended to other ranges of emotions. This challenges pure associative learning accounts, as young infants are exposed to many positive emotions and facial expressions from early on; presumably when they smile, they are copied/mimicked by their carers significantly more than when they are sad or cry.

*The role of ToM:* Cognitive empathy is generally assessed by asking participants to attribute others' emotions, based on a story or facial expression (see for example Knafo et al., 2009). While these tasks have been extensively used with school‐age children, just a few studies used them with toddlers. Is it because this empathy component indeed develops only later? Or because measuring cognitive empathy at this age when toddlers' expressive skills are still limited is methodologically challenging? Longitudinal studies might be the key to capture whether there are skills that from infancy could promote the maturation of cognitive empathy earlier than thought so far. There is evidence that the development of cognitive empathy in young children is associated with the development of theory of mind (ToM) (Bensalah et al., 2016), the ability to take other's perspective (Frith & Frith, 2005), and an extensive literature links empathy and ToM in adults (Preckel et al., 2018). As ToM is known to develop at around 4–5 years (Saxe, 2013), even though its precursors (i.e. attribution of false belief) have been detected by the 2nd year of life (Baillargeon et al., 2010; Southgate et al., 2007, but see also Baillargeon et al., 2018), one may hypothesise that cognitive empathy arises at this age too. Advancing new methods and tasks to investigate cognitive empathy with toddlers will elucidate if indicators of cognitive empathy cannot be identified earlier than the 4th year of life, which would rule out the *foundational cognitive empathy model*.

*Neural mechanisms:* To our knowledge there are only two neuroimaging studies that tested neural correlates of empathy in toddlers, likely due to the fact that children at this age struggle to stay still for long with the equipment on. In one study using electroencephalography (EEG), 3‐to‐5‐year‐olds showed a greater neuronal response (i.e. N200) to painful rather than neutral stimuli, with a greater effect on brain components associated in adults with affective rather than cognitive empathy (i.e. greater right frontal activation) (Decety et al., 2018). This is most consistent with the *foundational affective empathy model*. In a different study using functional near‐infrared spectroscopy (fNIRS), 4‐to‐8‐year‐olds activated both the medial and the dorsal orbitofrontal cortex during empathic scenarios with both affective and cognitive details indifferently, with older children showing greater activation in medial and dorsal orbitofrontal cortex and inferior frontal gyrus for emotional empathy than younger ones (Brink et al., 2011). This work did not find different networks in support of cognitive and affective empathy as are seen in adults. Inconsistent with both models proposed above, this study seems to suggest that the two empathic components are unitary early, at least at the neural level. However, this work assessed neural underpinnings of cognitive empathy by using scenarios representing situations not emotionally salient and more related to ToM/cognitive reasoning. Instead, we believe that neuroimaging studies should use emotionally salient scenarios to investigate both affective and cognitive empathy. Future studies are needed to elucidate whether brain regions for different empathic components are not yet specialised in childhood. In particular, fNIRS (that has a better spatial resolution than EEG) could be leveraged to explore networks specialisation for affective and cognitive empathy during toddlerhood. Finding neural activations stronger for affective rather than cognitive empathy in early toddlerhood would provide supporting evidence for the affective empathy foundational for the cognitive one. One the contrary, if patterns are reversed, we might think that cognitive empathy arises earlier than the affective one.

*Scaffolding:* During observations in the nursery, the teachers' role seemed to be a crucial scaffold to develop proper empathic reactions, and to use abstract and feeling words. In the nursery, several episodes of children approaching and staring at a classmate crying happened right after a teacher approached the child in distress. Moreover, there was one episode where the teacher hugged a child crying and a classmate did the same, following her example. It is well established that imitation is fundamental from early on to acquire social skills (i.e. social learning, Bandura, 1962), but its role in empathy development or in potential gender differences in the balance between cognitive and affective empathy (Volbrecht et al., 2007) has not been fully defined. Further, caregiver language is also important. In previous work 2‐year‐olds whose mothers made references to abstract concepts, such as needs, intentions, and desires, were lower in aggression‐related behaviours (Garner & Dunsmore, 2011). When attending a crying doll, 2‐year‐old toddlers whose parents commented about the crying baby labelled doll's emotion, and toddlers whose parent talked about helping the doll showed more empathic concern (McHarg et al., 2019). It is well recognised that parents and other carers in promoting the development of empathy (Tong et al., 2012). Consistent with this, it has been shown that high levels of synchrony during parent‐infant interactions and a secure attachment style foster an appropriate development of empathy (for example see Feldman, 2007; Mikulincer et al., 2001). It has to be noted though that there has been little emphasis on how infants transition skills learnt during the interactions with their carer to the interactions with their peers. Moreover, we know very little about how partner familiarity affects toddlers' empathic reactions; it is plausible to think that seeing a classmate whom the child had few interactions with in distress will elicit less empathy than seeing a carer in pain. Further, from 3 years the amount of pretend play and the use of abstract words, which helps the child taking someone else's perspective, significantly increased compared to before in both our observations and the broader literature (Howes & Matheson, 1992; Jaggy et al., 2020; Vallotton & Ayoub, 2011). Episodes of “staring” might reflect periods of attention that provide opportunities for learning when caregivers explain a child's emotions and model appropriate caring behaviour. A significant role for language and mimicry may provide partial support for the *foundational cognitive model*, because it suggests that early empathic behaviour is driven primarily by cognitive processes and less by direct sharing of another's feelings.

*New methods:* There is a clear need to develop methods for assessing components of empathy that do not rely on verbal or reasoning skills, because otherwise these will confound assessments of developmental ordering. Moreover, the study of empathy needs to go beyond behavioural tests and observations, as with these methods we can just assess what toddlers *show*, but not what they really *feel*. However, the traditional settings currently in use in most labs are inappropriate for assessing toddlers, who struggle to comply with strict testing rules and might exhibit unnatural behaviours (see Box 4 for the methods used so far to assess affective and cognitive empathy). To explore mechanisms supporting empathy development, while allowing toddlers to freely move around, labs should adopt the use of wearable neuroimaging methods, such as fNIRS and EEG (Pinti et al., 2021), and measures of physiological arousal (i.e. heart‐rate and perspiration). These wearable methods can easily be brought outside the neuroscience labs, and implemented in homes or nurseries, where we can study dynamics related to cooperation and interactions involving more than one child at the time. While most of the studies performed so far assessed toddlers' empathy towards adults, testing toddler‐toddler interactions will represent more closely what they experience daily at nursery or at the playground. Toddlers may display more empathy with their peers as they see them as similar to themselves. Moreover, the other toddler will respond back naturally without consciously trying to scaffold or improving empathy in the child, as a carer would likely do. Dissecting neural underpinnings of different empathic components in a naturalistic set‐up is challenging too. One possible way to do this is detecting activations of brain regions known to be engaged by cognitive or affective empathy and identify which event of the social interaction triggered these activations.

**FIGURE 4:**
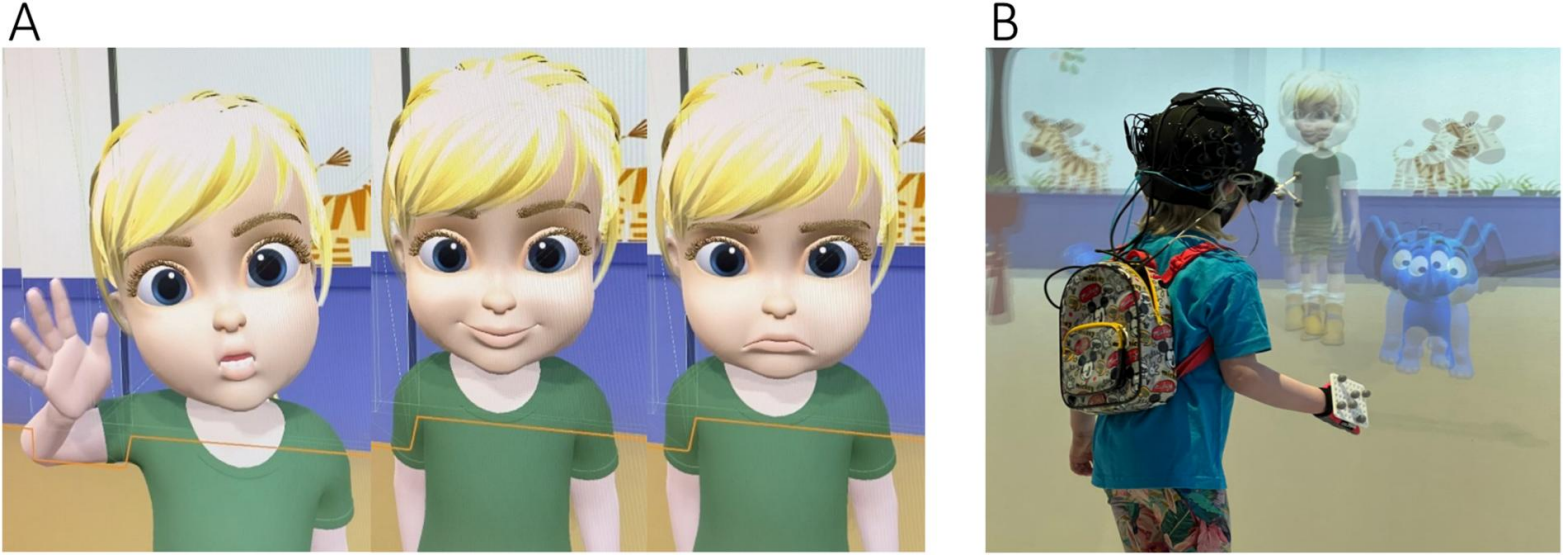
(A) A toddler‐like avatar in a virtual reality (VR) set‐up waiving, smiling and being sad. (B) A 5‐year‐old wearing wearable fNIRS playing with the avatar in the VR set‐up of the Birkbeck ToddlerLab. Image courtesy of Dr. Paola Pinti and Dr. Nadine Aburumman.

Interestingly, wearable neuroimaging can be implemented in immersive virtual reality (VR). This novel technology can be leveraged to assess toddlers within a realistic but controlled scenarios, such as a playground or a nursery class. Taking advantage of the immersive features of the VR to study social development, toddlers could be able to feel the first‐person experience that characterises empathy, whilst freely moving (Figure 4; Bulgarelli et al., 2022). This cutting‐edge method will allow to have total control over the experimental variables, which is not possible in live‐interaction studies, and it might be the key to successfully understand the toddlerhood world.

**FIGURE 5:**
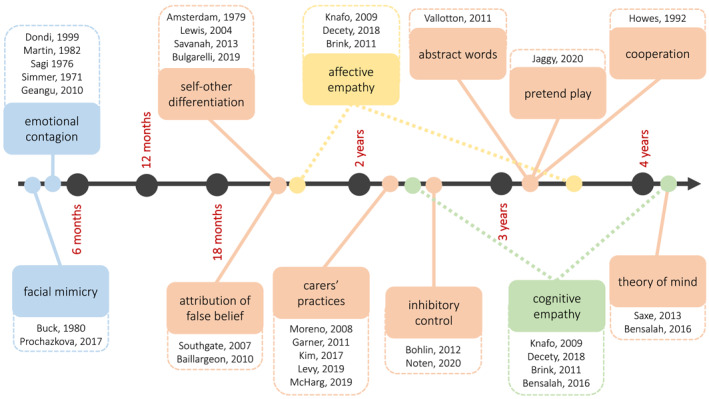
Timeline of the development of empathy components and other skills that might influence them. Affective empathy is represented in yellow, cognitive empathy in green, precursors of empathy in blue and other skills in orange. Dotted lines of affective and cognitive empathy indicate the open debate of when the two components mature.

Figure 5 shows a timeline of when precursors and components of empathy, and other skills that might influence its development, mature.

### Early markers of atypical development of empathy in toddlerhood

**FIGURE 6:**
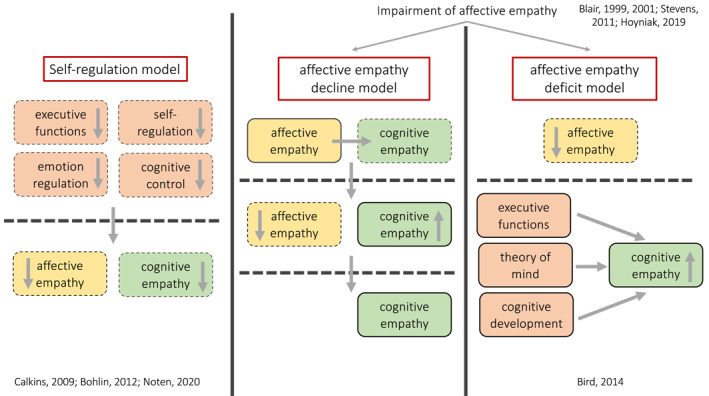
Graphical representation of the three models explaining mechanisms underlying the atypical development of empathy. Affective empathy is represented in yellow, cognitive empathy in green and other skills in orange. Skills that are low or decreasing are marked with a dotted line, while skills that are established or increasing are marked with a straight line.

Poor or absent empathic skills lead to severe difficulties in social interactions, and quite often, if this aspect considerably compromises the child's social life, result in a diagnosis of antisocial behaviour or conduct disorder (Calkins & Keane, 2009; Viding & McCrory, 2018). While most of the diagnoses are formalised in late childhood (Crowe & Blair, 2008), assessments of poor empathic skills can be performed from early childhood (Ezpeleta et al., 2013; Kimonis et al., 2016). Indeed, parents' rating of their own child's empathy were found to be negatively associated with CU traits from 3 years of age (Dadds et al., 2009). Early diagnosis, or at least early identification of atypical empathic reactions, could support efficient and early interventions. Hereafter we focused on summarising the few studies on early signs of empathic impairments in toddlerhood, as several other reviews extensively covered atypical empathy in childhood (see for example Viding & McCrory, 2018). We identified three main theoretical models supported by empirical evidence that suggest different mechanisms underlying poor empathy: (i) regulatory deficits model; (ii) affective empathy decline model; iii) affective empathy deficit model (Figure 6).

A first model (*regulatory deficit model*) proposed that a common pattern of regulatory deficits underlies the atypical development of empathy. This framework seems to be consistent with the idea of cognitive empathy developing prior the affective one (*foundational cognitive empathy model*), as it suggests that cognitive skills promote the emergence of empathy. However, to data there is no empirical evidence supporting this hypothesis. In this framework, impaired emotion regulation, self‐regulation and cognitive control as early as at 2 years might be linked to low levels of empathy and behavioural problems throughout development (Calkins & Keane, 2009). Consistent with this, inhibitory control was found to moderate the relation between heart rate responses during an empathy task and aggression in 30‐month‐olds, with a negative association between heart rate response and aggression when inhibitory control was high, but a positive association when inhibitory control was low, suggesting that high levels of empathy and inhibitory control protect children from being aggressive (Noten et al., 2020). There is also evidence that inhibitory control in toddlerhood predicts externalising problems, known to be related to poor empathy (Cooper et al., 2020), in late childhood (Bohlin et al., 2012). Future research on empathy could benefit from exploring further the role of inhibitory control as a mediating factor of empathy development. In fact, whether inhibitory control has a direct or indirect (through suppressing aggressive behaviours) role on empathy is still unclear, but could inform interventions focused on improving inhibition skills.

The second and the third models both build on the evidence that only the affective component of empathy, and not the cognitive one, is impaired in psychopaths or adults with severe antisocial behaviours (Blair, 2005; but see also Brook & Kosson, 2013). Both the second and the third theoretical models suggest that affective impairments in individuals with high levels of CU traits might change over the life span (Frick & Kemp, 2021). In fact, there is evidence that young children with high levels of CU traits are deficient in both affective and cognitive empathy. For example, it has been shown that children with impaired empathy struggled to label correctly emotionally connoted facial expressions (Stevens et al., 2001), especially fear (Blair et al., 2001), indicating impaired cognitive empathy. Electrodermal responses to distress and threatening cues were found to be significantly lower in children with emotional difficulties compared to a control group (Blair, 1999), and neural index of facial emotion processing measured with EEG was lower in children with high levels of CU traits than in typically developing 3‐to‐5‐year‐olds (Hoyniak et al., 2019), indicating impaired affective empathy. Interestingly, over time children with poor empathic skills might acquire the ability to understand and predict others' feelings and mental states. But what is the mechanism that favours the development of cognitive empathy in children with poor affective empathy? If the *foundational affective model* (Decety et al., 2018) and the idea of a natural predisposition to react to others' emotions are correct (Geangu et al., 2011), one may hypothesise that in children with high levels of CU traits affective empathy first develops, as it does in all infants, and then gradually decays with age whilst cognitive skills continue to improve (*affective empathy decline model*). This is consistent with the idea of a necessary interaction of a natural predisposition towards others with a subsequent validation and reinforcement of these social reactions from the surrounding environment when looking at these aspects of infant development. However, to date there is no empirical evidence showing indicators of affective empathy decreasing with age in toddlers (due also to the fact that most of the studies on children with high levels of CU traits assessed school‐age children or adolescents).

Another view is that individuals lacking affective empathy might have had fewer experiences of distress in infancy, as for example, they seek for less eye‐contact, and therefore less opportunity to learn which cues indicate distress in others (Bird & Viding, 2014). This model (*affective empathy deficit model*) might suggest then that children with impaired empathic skills might have never developed the affective component, and therefore they develop cognitive empathy through different routes (i.e. general cognitive development, development of executive functions and ToM), rather than building on affective empathy (absence of affective empathy and maturation of cognitive empathy). Alternatively, consistent with the *foundational cognitive model*, these children might develop cognitive empathy first, but they are not able to transition what they learnt from others' feelings to share those feelings in an other‐oriented way.

## CONCLUSION AND FUTURE DIRECTIONS

This work reviewed current knowledge on the development of empathy in toddlerhood by integrating evidence from empirical studies with observations of toddlers in their own habitat, a nursery. Understanding how empathy develops and what goes awry in some children with poor empathic reactions is crucial to design early interventions and therefore reduce severe diagnoses in late childhood.

Emotional contagion, as a possible primitive form of empathy, has been widely documented in newborns and infant studies. This behaviour was observed at the nursery too in younger toddlers, but less frequently than indifference to a classmate crying. This might challenge the importance of this behaviour and the timescale of emergence of affective empathy. Further, watchful interest in another toddler crying is a more mature behaviour that could reflect personal distress (affective empathy) but could also reflect an attempt to understand why the other child cries and a window for a parent or carer to provide verbal or physical scaffolding (cognitive empathy). Reviewing the (limited) current studies on empathy in toddlers reveals that the dynamics of development of the two empathic components and the precise parent or caregiver behaviours that are most important in scaffolding them remains unclear. Further, the nature of the mechanisms that underpin atypical empathy and whether it is an early lack of affective or cognitive empathy at the route of later difficulties is critical to designing more effective early interventions. We have highlighted several fundamental open questions that we believe the field should address to better understand how empathy and all its facets mature in toddlerhood (Box 5). Throughout this work, we also recommended the use of cutting‐edge methods, such as wearable neuroimaging and VR, to more efficiently and naturally assess empathy in toddlerhood. We hope that the field of social development can benefit from this work, and use it as a starting point to further investigate empathy development and improve outcomes for children with high levels of CU traits.

**BOX 5 Key research questions for next‐generation**

- Can we use longitudinal studies to elucidate the link between early facial mimicry and later empathic skills?
- What is the mechanism that links emotional contagion to empathy? Self‐other differentiation seems to be necessary but not sufficient to develop proper empathic reactions. What are other factors that might influence this transition and how could they be assessed using naturalistic neuroimaging?
- Are the neural underpinnings of cognitive and affective empathy specialised in toddlerhood? Which emerges first?
- Can we leverage new methods and tasks to identify evidence of cognitive empathy before the 4th year of life?
- Can we provide empirical evidence of which empathic component matures first? Does other—oriented affective empathy emerge from emotional contagion, or through cognitive scaffolding?
- Do we need to develop new measures and terminology to clearly distinguish shared feelings that represent other‐oriented affective empathy from those that represent self‐oriented personal distress throughout development?
- Nature versus nurture: how does predisposition to empathy interact with copying empathic behaviours from others? Are gender differences in empathy established from toddlerhood? Could different parenting practice to boys and girls influence gender differences in empathy?
- Does greater exposure to cooperation, pretend play or abstract words early in life promote higher levels of empathy later in the development? What are the mechanisms that link these aspects together?
- Could we use virtual‐reality to train appropriate empathic reactions in toddlers?
- What is the role of inhibition in the development of empathy?
- Through which mechanism do children with poor affective empathy develop cognitive empathy?
- How does having poor empathy limits social learning from others during early development?

## AUTHOR CONTRIBUTIONS

**Chiara Bulgarelli**: conceptualisation, data curation, funding acquisition, investigation, project administration, visualisation, writing—original draft preparation. **Emily J. H. Jones:** conceptualisation, supervision, writing—review and editing.

## CONFLICTS OF INTEREST

Emily J. H. Jones is a Joint Editor for JCPP *Advances*. Chiara Bulgarelli has declared that they have no competing or potential conflicts of interest.

## ETHICAL CONSIDERATIONS

Ethical approval for this study was given by the Ethics Committee of the Department of Psychological Sciences, Birkbeck, University of London (No. 2122056).

## Supporting information

Supporting Information S1

## Data Availability

Data sharing is not applicable to this article as no new data were created or analysed in this study. Data of observations of children in the nursery are described in full in the article.

## ENDNOTES

^1^
Note that most of the times antisocial behaviour and conduct disorder (or problems) are used interchangeably. However, in clinic, conduct disorder is considered more severe than antisocial behaviour (NICE and SCIE, 2017).

^2^
There might be some confusion around the use of the terms affective empathy and sympathy. While affective empathy is experiencing similar emotions to someone else's emotions, sympathy is experiencing concern and sorrow (i.e. compassion, pity, warm‐heartedness) in response to negative impacts on other's wellbeing (Maibom, 2009).

^3^
Self‐other distinction in infants is assessed using the mirror self‐recognition task (MSR) (Amsterdam, 1972). At 18 months roughly 50% of the infants recognise themselves in the mirror and by 24 months all infants are generally successful at this task. Despite the criticism around this task (see Mitchell, 1993), it is still considered the dominant measure to test the development of self in infancy, assessing something more than mere self‐recognition. In fact, infants' successful performance at the MSR task has been related to personal pronouns use (Lewis & Ramsay, 2004), symbolic representation of one's own body (Savanah, 2013), and to a network of brain regions associated with abstract self‐processing (Bulgarelli, Blasi, de Klerk, et al., 2019).
