# Supplementary material for: The typical and atypical development of empathy: How big is the gap from lab to field?
Source: JCPP Adv. 2023 Jan 24;3(1):e12136. doi: 10.1002/jcv2.12136 (PMC10241450; doi:10.1002/jcv2.12136)
Supplement: Supplementary file 1 — Supporting Information S1 [file JCV2-3-e12136-s001.docx]

**Supporting Information**

**Participants and procedure of the naturalistic observations in a nursery class**

Observations were performed in a nursery in North London. The study was approved by the Ethical Committee of the Department of Psychology, Birkbeck, University of London. An opt-out consent form was sent out in advance to all the parents of the children attending the nursery, and no-one withdrew their child from the observation sessions. Observations took place for 2 hours (10.00AM-12.00PM) for 3 mornings a week for 3 consecutive weeks in April/May 2022, for a total of 21 hours. The observations focused on 2 classes, 2-to-3-year-olds (max capacity = 24 children), and 3-to-4-year-olds (max capacity = 34 children). The nursery had also an outside playground where toddlers of both classes mixed. The headmaster and the teachers of the nursery were instructed on not changing anything about their routine and activities with the children during the observation sessions. The experimenter sat on different corners of the class or outside in the playground, without interfering with children’s dynamics (beyond responding if approached by them, e.g. smiling, or saying hello). The experimenter wrote notes on toddler-toddler and toddler-teacher interactions, focusing particularly on emotionally salient exchanges. No names were noted down, but only the sex and the age of the child observed (e.g. in the experimenter’s notes, M(3) refers to a 3-year-old boy).
